# Effect of In Vitro Ruminal pH on Zearalenone Degradation and Interaction with Other Mycotoxins in a Static Gastrointestinal Model

**DOI:** 10.3390/toxins17010013

**Published:** 2024-12-30

**Authors:** Rimvydas Falkauskas, Jurgita Jovaišienė, Gintarė Vaičiulienė, Sigita Kerzienė, Ingrida Jacevičienė, Eugenijus Jacevičius, Inga Jarmalaitė, Marija Ivaškienė, Gintaras Daunoras, Rasa Želvytė, Violeta Baliukonienė

**Affiliations:** 1 Department of Food Safety and Quality, Faculty of Veterinary, Lithuanian University of Health Sciences, Tilzes Str. 18, LT-47181 Kaunas, Lithuania; jurgita.jovaisiene@lsmuni.lt (J.J.); violeta.baliukoniene@lsmuni.lt (V.B.); 2 National Food and Veterinary Risk Assessment Institute, J. Kairiukscio Str. 10, LT-08409 Vilnius, Lithuania; ingrida.jaceviciene@nmvrvi.lt (I.J.); eugenijus.jacevicius@nmvrvi.lt (E.J.); inga.jarmalaite@nmvrvi.lt (I.J.); 3 Animal Reproduction Laboratory, Large Animal Clinic, Veterinary Academy, Lithuanian University of Health Sciences, Tilzes Str. 18, LT-47181 Kaunas, Lithuania; gintare.vaiciuliene@lsmuni.lt; 4 Department of Animal Breeding, Faculty of Animal Science, Lithuanian University of Health Sciences, Tilzes Str. 18, LT-47181 Kaunas, Lithuania; sigita.kerziene@lsmuni.lt; 5 Dr. L. Kriaučeliūnas Small Animal Clinic, Faculty of Veterinary Medicine, Lithuanian University of Health Sciences, LT-47181 Kaunas, Lithuania; marija.ivaskiene@lsmu.lt (M.I.); gintaras.daunoras@lsmu.lt (G.D.); 6 The Research Center of Digestive Physiology and Pathology, Lithuanian University of Health Sciences, Tilzes Str. 18, LT-47181 Kaunas, Lithuania; rasa.zelvyte@lsmu.lt

**Keywords:** zearalenone, ruminal, variation, alfa-zearalenol, beta-zearalenol

## Abstract

The degradation of zearalenone (ZEN) in the rumen of dairy cows is influenced by rumen pH, which is a key factor affecting this process. The aim of this study was to investigate the variation of ZEN in interaction with other mycotoxins at different ruminal pH environments (physiological (pH 6.5) and acidic (pH 5.5)) using an in vitro rumen model. Rumen fluid was collected from the caudoventral part of the rumen of cows using a pharyngeal–esophageal probe. To determine the changes in different mycotoxins (ZEN; AFLB_1_; DON; T-2) in the rumen of cows, a model rumen system was used, and mycotoxins concentrations were detected by HPLC. The study found that at pH 6.5, ZEN alone and in combination with other mycotoxins (DON; T-2; AFLB_1_) significantly (*p* < 0.05) reduced ZEN levels compared to the rumen environment at pH 5.5. It was observed that α-zearalenol (α-ZEL) and β-zearalenol (β-ZEL) concentrations were generally higher at a rumen pH of 6.5 compared to pH 5.5, averaging 47.09 µg/L and 35.23 µg/L, respectively. Additionally, the frequency of detection for both α-ZEL and β-ZEL was greater at pH 6.5 than at pH 5.5. A comparison of α-ZEL concentrations in rumen samples at pH 5.5 showed a 20% increase from the 6th to the 9th hour of the test, while β-ZEL levels remained unchanged over the same period.

## 1. Introduction

Zearalenone (ZEN) is a nonsteroidal estrogenic mycotoxin primarily produced by *Fusarium* fungi, which contaminates various agricultural commodities, including maize and other cereals [1]. Due to its structural similarity to estrogen, ZEN can disrupt endocrine functions in livestock, leading to reproductive and developmental disorders [2]. The presence of ZEN in animal feed is of particular concern for ruminants, as the rumen environment can biotransform mycotoxins into metabolites with either reduced or increased toxicity [3].

Globally, ZEN contamination is a significant concern in livestock feed, with prevalence rates varying widely depending on geographic region, climate conditions, and storage practices. For instance, a study reported ZEN contamination in 92.3% of feed ingredient samples in China, indicating a high prevalence in certain regions [4]. Another survey found that ZEN contamination levels in animal feed can reach up to 42.5% in various livestock feeds. This widespread occurrence poses a threat to livestock health and productivity. Therefore, understanding the factors influencing ZEN degradation and the formation of its metabolites is crucial for developing effective mitigation strategies [5].

The rumen is a complex anaerobic fermentation chamber where microbial consortia play a crucial role in the metabolism of various dietary components, including mycotoxins. The efficiency of microbial detoxification processes depends on various factors, such as diet composition, microbial community structure, and ruminal pH [6]. Among these, ruminal pH significantly influences microbial activity and, consequently, the biotransformation pathways of toxins. Variations in ruminal pH, which can range from 5.5 to 7.0, can alter the enzymatic activity of microbes, affecting the detoxification efficiency of ZEN and other co-occurring mycotoxins [7].

Additionally, ZEN rarely occurs in isolation; it is often found alongside other mycotoxins, such as deoxynivalenol (DON) and fumonisins, in contaminated feed [8]. The interaction between ZEN and these co-occurring mycotoxins can result in synergistic or antagonistic effects, complicating the risk assessment and management strategies for animal health. Previous studies have shown that the simultaneous presence of multiple mycotoxins can impact the metabolism of each compound in the rumen, leading to unpredictable biotransformation outcomes [9].

The novelty of this study lies in the investigation of multiple feed contaminations with combinations of mycotoxins (ZEN; AFLB_1_; DON; T-2). The effects on animal health and the mode of action can be cumulative, antagonistic, or synergistic. This means that overall toxicity is not merely the sum of individual mycotoxin toxicities but can also be amplified, making the evaluation of a single toxin insufficient to fully assess the risks associated with specific feed or feed materials. Greater attention must be paid to the cumulative effects of mycotoxins, as feed is rarely contaminated by only one mycotoxin. Therefore, in our study, we aimed not only to assess the impact of ruminal pH on mycotoxin degradation but also to evaluate the cumulative effects of mycotoxins on both the formation of ZEN metabolites and the reduction of mycotoxins.

Given the complexity of mycotoxin interactions and the variability of ruminal conditions, in vitro, rumen models provide a controlled environment to study the biotransformation of mycotoxins under varying pH levels (simulating physiological (pH 6.5) and acidic (pH 5.5). These models simulate the dynamic rumen environment and allow for precise manipulation of conditions, making them an effective tool for understanding the fate of ZEN and its interaction with other mycotoxins [10].

The hypothesis of this study is that not only the cumulative effects of mycotoxins may influence the degradation of individual mycotoxins and the formation of ZEN metabolites, but also that these processes are affected when cows develop acidosis.

The aim of this study was to evaluate the variation of zearalenone and other mycotoxins in combination at different ruminal pH environments using the static gastrointestinal model in vitro.

## 2. Results

### 2.1. The Effect of Rumen pH Environment on the Concentration Changes of Mycotoxins

The study found that ZEN and ZEN in combination with other mycotoxins (DON, T-2 toxin (T-2), aflatoxin B_1_ (AFLB_1_)) showed a significantly higher reduction (*p* < 0.05) at pH 6.5 compared to rumen pH 5.5 (Table 1). The highest percentage reduction of ZEN was observed in combination with DON after 9 h (100%, *p* < 0.05). At rumen pH 5.5, the greatest reduction in ZEN (46.6 ± 0.8%, *p* < 0.05) occurred in combination with AFLB_1_, DON, and T-2, also after 9 h. A statistically significant the percentage reduction of ZEN was observed for all combinations of mycotoxins.

A 96.4% reduction in ZEN combined with DON was detected at pH 6.5 after 9 h (*p* < 0.05), compared to the reduction observed after 3 h at the same pH. After 9 h, the percentage reduction of ZEN at pH 6.5 was 61.6% (*p* < 0.05) higher than the reduction observed at pH 5.5 after 9 h.

The percentage reduction of ZEN in combination with T-2 increased by 43.5% (*p* < 0.05) at pH 6.5 after 9 h compared to 3 h. At pH 5.5, the percentage reduction of ZEN in combination with T-2 increased by 22% after 9 h compared to 3 h. The reduction in ZEN at pH 6.5 was 27.1% (*p* < 0.05) higher compared to the reduction after 9 h at pH 5.5.

The percentage reduction of ZEN in combination with AFLB_1_, DON, and T-2 increased by 24.4% (*p* < 0.05) after 9 h compared to 3 h at pH 6.5. At pH 5.5, the reduction increased by 16.2% (*p* < 0.05) after 9 h compared to 3 h. After 9 h, the percentage reduction of ZEN at pH 6.5 was 2.4% (*p* < 0.05) higher compared to the reduction at pH 5.5.

Summarizing the data in Table 1, after 3 h, the lowest percentage reduction in ZEN at rumen pH 6.5 was observed in combination with DON and T-2, while after 6 and 9 h, the lowest reduction occurred in combination with AFLB_1_, DON, and T-2.

The percentage reduction of T-2, AFLB_1_, and DON in combination with ZEN was determined in vitro using a rumen model system (Table 2). The highest percentage reduction after 9 h was observed in the combination of DON with ZEN, while the lowest was found in the combination of T-2, AFLB_1_, DON, and ZEN.

It was found that after 9 h, the percentage reduction of AFLB_1_ in the investigated combinations at pH 6.5 was 0.5—% higher (*p* < 0.05) compared to 9 h at pH 5.5. The percentage reduction of DON in combinations after 9 h at pH 6.5 was 1.7% higher (*p* < 0.05) than at pH 5.5. Similarly, the percentage reduction of T-2 in combinations after 9 h at pH 6.5 was 18.8% higher (*p* < 0.05) compared to the reduction observed after 9 h at pH 5.5.

### 2.2. The Effect of Rumen pH Environment on the Concentration Changes of Certain ZEN Metabolites

**Figure 1:**
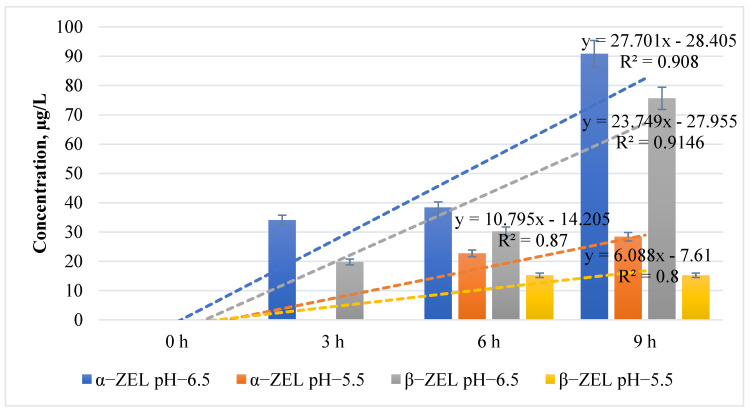
Change in α−ZEL and β−ZEL concentrations in physiological and acidic rumen conditions of dairy cows in vitro considering the duration of exposure.

ZEN metabolites α-ZEL and β-ZEL were also detected in vitro in the rumen model system (Figure 1). It was found that at pH 6.5 of the rumen, α-ZEL and β-ZEL concentrations were found to be on average higher compared to pH 5.5 at 47.09 µg/L and 35.23 µg/L, respectively. Also, the detection frequency of α-ZEL and β-ZEL was higher at pH 6.5 compared to pH 5.5. The comparison of the change in α-ZEL concentrations found in the rumen pH 6.5 samples after 9 h of the study with 3 h of the study showed an increase of 56.81% and β-ZEL an increase of 55.98%. The comparison of the change in α-ZEL concentrations in the rumen pH 5.5 samples after the 9th hour of the test with the 6th hour of the test showed a 20% increase, but no change in β-ZEL. It was also found that in the pH 5.5 samples from the rumen, the concentrations of α-ZEL and β-ZEL were below the detection limit of the test method up to the 3rd hour of the study (LOD) (α-ZEL—0.04 μg/L; β-ZEL—0.05 μg/L).

## 3. Discussion

Ruminant rumen microbiota provides the first line of defense. The microbiota allows some mycotoxins to be broken down into less toxic compounds [11]. Despite this breakdown of mycotoxins, the rumen microbiota can convert mycotoxins (e.g., ZEN) into metabolites with more potent toxic effects than the mycotoxin itself [12].

The scientific literature points out that lowering the pH of the large rumen below 6.3 weakens the activity of bacteria involved in the breakdown of mycotoxins. A decrease in pH below 6 may alter the ratio of bacteria involved in mycotoxin degradation and thus influence the reduced mycotoxin degradation in the rumen [13,14,15,16]. Placinta et al. [16] suggest that a reduced pH of the rumen alters the bacterial ecosystem. Therefore, if an animal was diagnosed with acidosis, the ability of the rumen microbiota to detoxify mycotoxins would be severely reduced.

We found that ZEN and ZEN in combination with the other tested mycotoxins showed lower changes in vitro at rumen pH 5.5, which agrees with the results obtained by researchers [13,14], who suggest that the acidic environment of the rumen negatively influences the detoxification process of mycotoxins. This is also supported by another study in which the pH of the rumen during the first 6 h did not influence the change in ZEN [17].

Our findings indicate that the combination of ZEN with T-2 toxin resulted in the smallest reduction of ZEN in the rumen’s physiological environment (pH 6.5). It is hypothesized that the T-2 toxin may exert an antagonistic effect on ZEN degradation, potentially due to its chemical structure. Both our data and those reported by other researchers [18,19] demonstrate a gradual decline in ZEN concentration within rumen fluid over time. This reduction may be attributed to the activity of protozoa in the rumen, which possess significant detoxification capacity.

It is important to emphasize that mycotoxin metabolism in the rumen does not necessarily result in complete detoxification of these compounds [20]. The results of our study confirm this, as most of the combinations of mycotoxins studied with ZEN showed a percentage reduction of about 50% after 9 h, indicating that about half of the mycotoxins studied can enter the intestines. On the other hand, Debevere et al. [17] reported a 50% reduction in ZEN in the rumen fluid only after 48 h.

An in vitro study demonstrated that the percentage change in DON was influenced by the rumen’s pH environment. Similar findings were reported by He et al. [21], who concluded that DON degradation is affected by both pH levels and microbiota activity. The researchers suggested that lowering the pH to 5.2 inhibits DON degradation [21]. This conclusion is consistent with a 2020 study showing that low rumen pH impacts the degradation of both DON and ZEN in vitro [20]. However, our findings indicate that a reduction in rumen pH to 5.5 after 9 h does not correspond to a decrease in DON degradation. The concentration of DON was reduced by half after 9 h, suggesting no significant impact of pH on its breakdown.

We observed that the percentage change in AFLB_1_ was influenced by the rumen’s pH environment when AFLB_1_ was combined with ZEN, but not when combined with DON/ZEN/T-2. Despite this, we found a consistent decrease in the percentage change of AFLB_1_ over time, regardless of pH. This decrease is likely due to the sustained activity of the rumen microbiota. Jiang et al. [22] also proposed that rumen microbiota exhibit a high capacity for AFLB_1_ metabolism. However, our results differ from those of Kiessling et al. [11], who found that rumen pH did not affect AFLB_1_ reduction in a similar mycotoxin combination.

In our in vitro study, we observed that the percentage change in T-2 was influenced by rumen pH when combined with AFLB_1_/DON/ZEN, but not when combined solely with ZEN. These results partially align with the findings of Debevere et al. [17] and Li et al. [23], who suggest that rumen microbiota is capable of detoxifying T-2 toxin, though factors such as an acidic rumen environment can impair this detoxification process. In our study, the overall reduction of T-2 in the pH 6.5 environment was 5.75% higher in combination with ZEN and 23% higher in combination with AFLB_1_/DON/ZEN compared to the pH 5.5 environment, further supporting the assertion that pH can influence T-2 levels in the rumen.

In our model system of the rumen, we detected α-ZEL and β-ZEL in vitro. This confirms the claims of researchers [11,17] that the primary degradation of ZEN by the rumen microflora takes place. In our study, higher concentrations of α-ZEL than β-ZEL were detected by an average of 25%.

Similar findings have been reported by other researchers in an in vitro study, which demonstrated that ZEN degradation in the rumen tends to favor the formation of α-ZEL over β-ZEL [11]. In our study, we also observed that pH significantly influenced the levels of α-ZEL and β-ZEL. On average, α-ZEL concentrations were 45% higher at rumen pH 6.5 compared to pH 5.5, while β-ZEL concentrations were 56% higher at pH 6.5 than at pH 5.5. Belgian researchers [20] obtained comparable results regarding ZEN metabolites at different rumen pH levels. They found that at pH 6.8, the concentration of α-ZEL was approximately 12.5% higher than at pH 5.8. Similarly, β-ZEL levels at pH 6.8 were up to 6% higher than those observed at pH 5.8.

In our in vitro rumen model system, we monitored the variation in ZEN metabolites for up to 9 h. At rumen pH 5.5, neither α-ZEL nor β-ZEL was detected during the first 3 h. We hypothesize that in dairy cows experiencing acidosis, ZEN degradation within 3 h post-feeding may be minimal or absent. However, our study also revealed a 3.6-fold increase in α-ZEL after 9 h, indicating a potentially undesirable effect in dairy cows.

## 4. Conclusions

The results of this study show that analyzing mycotoxin degradation in the rumen model in vitro, under physiological (pH 6.5) and acidic (pH 5.5) conditions, the highest reductions of ZEN, AFLB_1_, DON, and T-2 in selected combinations were observed in the pH 6.5 environment after 9 h. Also, in both in vitro pH environments of the rumen model, the highest concentrations of α-ZEL and β-ZEL were detected after 9 h, while at pH 5.5 these mycotoxins were detected at levels below the limit of detection after 3 h. To optimize ruminal pH and minimize mycotoxin toxicity, we recommend maintaining a ruminal pH of approximately 6.5. To achieve this, ensure the diet includes an adequate amount of structural fiber, limit the proportion of concentrated feeds to no more than 60% of the total dry matter intake, and provide constant access to clean water. Periodically monitor ruminal pH using ruminometers or other available methods, and continuously track mycotoxin concentrations in the feed.

## 5. Materials and Methods

### 5.1. Rumen Fluid, Mycotoxins, Chemicals and Reagents

Rumen fluid was collected from the caudoventral part of the rumen of dairy cows using a pharyngealr–esophageal probe at least 2 h before morning feeding. Samples of rumen fluid were taken from clinically healthy study dairy cows. The collected rumen fluid was filtered through a 1 mm mesh sieve and the microbiota of the sampled contents was assessed using the infusoria. The in vitro rumen model system used rumen fluid with an infusoria count of at least 5.45 log_10_/mL. The collected rumen fluid was immediately transferred to the lab in thermos flasks before preparing the buffer–rumen fluid solution (see Section 5.2). The diet of the dairy cows can be found in Table 3.

Mycotoxins ZEN, AFLB_1_, DON, and T-2 were purchased from Sigma-Aldrich Chemie GmbH, Germany. Sodium bicarbonate, disodium hydrogen phosphate dodecahydrate, monopotassium phosphate, ammonium bicarbonate, magnesium chloride hexahydrate, and ethanol were purchased from Sigma–Aldrich Chemie GmbH, Germany. Carbon dioxide (CO_2_) was purchased from Linde, Lithuania.

Combinations of mycotoxins and their concentrations in vitro rumen model system were selected according to average concentrations of mycotoxins found in feed made in Lithuania.

### 5.2. Determination of Changes in Mycotoxin Concentrations in a Static Gastrointestinal Model In Vitro System in the Rumen of Dairy Cows

To determine the changes of different mycotoxins (ZEN, AFLB_1_, DON, T-2) in the rumen of cattle, a static gastrointestinal model in vitro system was created using a previous method with some modifications, as described by Debevere et al. [1].

The buffer solution for the model system was prepared for at least 12 h prior to the start of the test by adding 8.74 g sodium bicarbonate (NaHCO_3_), 3.58 g disodium hydrogen phosphate dodecahydrate (Na_2_HPO_4_-12H_2_O), 1.55 g monopotassium phosphate (KH_2_PO_4_), 1.00 g ammonium bicarbonate (NH_4_HCO_3_), and 0.124 g magnesium chloride hexahydrate (MgCl_2_-6H_2_O) to 1 liter of water of purity grade 2. The prepared buffer solution was saturated with CO_2_ and stored for 12 h at 39 °C.

Solutions of combinations of ZEN, DON, AFLB_1_, and T-2 were prepared by dissolving them in an ethanol/H_2_O (50/50, *v*/*v*) mixture. The in vitro concentrations used in the rumen model system were: ZEN—1000 µg/L DON—600 µg/L, AFLB_1_—7 µg/L, T-2—500 µg/L.

Two different pH environments were used in the static gastrointestinal model system, simulating physiological (pH = 6.5) and acidic (pH = 5.5) pH environments. All mycotoxin combinations used in the study were tested in five replicates.

A total of 1 L of buffer was mixed with 263.2 mL of the contents of the rumen before mixing the buffer solution with the mycotoxin combination mixture.

For the test, the contents of the rumen + buffer + mycotoxin combination mixtures were poured into separate incubators of the Ankom Daisy II (ANKOM Technology, Macedon, NY, USA) feed digestion system and kept at 39 ± 2 °C for 9 h. Throughout the study, the pH of all reaction solutions was pH = 6.5 or pH = 5.5 (depending on the experimental stage), adjustable as needed with 6 M of hydrochloric acid (HCl) (to simulate the physiological pH (pH = 6.5) and acidic (pH = 5.5) of the rumen). Changes in mycotoxin concentrations were recorded after 0 h, representing feed intake, 3 h and 6 h, representing intermediate times, and finally 9 h, indicating the feed’s passage from the rumen, because the feed in cow rumen can remain for up to 9 h before passing into the omasum and other compartments of the cow stomach.

### 5.3. Determination of Mycotoxin and ZEN Metabolites Concentrations

The concentrations of AFLB_1_ and ZEN were tested using high-performance liquid chromatography (HPLC) with a fluorescent detector (FLD) (Model LCMS-8060 Shimadzu Corporation, Kyoto, Japan). The concentration of DON was determined using HPLC with an ultraviolet detector (UV) (Model Sciex API 5000, McKinley Scientific, Sparta Township, NJ, USA). The T-2 toxin was determined using liquid chromatography–mass spectrometry (LC-MS).

Samples were extracted in distilled water for DON, in methanol–water (75:25 *v*/*v*) for AFLB1 and ZEA, and in methanol–water (60:40 *v*/*v*) for the T-2 toxin at constant mixing on a mechanical shaker (Phoenix Instrument RS-OS 20, Inc., Garbsen, Germany) for 60 min at 23 °C. After extraction, the samples were centrifuged at a relative centrifugal force (RCF) of 3468× *g* for 10 min (Centrifuge MPW-251, MPW, Warsaw, Poland). Later, the supernatants were filtered using PTFE syringe filters with pore diameters of 0.22 μm (Millex-GS, Millipore, Billerica, MA, USA) and diluted in phosphate-buffered saline (PBS). In the sample purification step, the extracts were passed through a multi mycotoxin immunoaffinity column 11 + Myco MS-PREPR (R-Biopharm AG, Darmstadt, Germany) according to the manufacturer’s recommendations. The prepared samples were subjected to high-performance liquid chromatography analysis. HPLC conditions for mycotoxins: column temperature for AFLB_1_, ZEN and DON: 30 °C, for T-2 toxin: 40 °C; mobile phase for ZEN: H_2_O/ACN/methanol (MeOH) (46/46/8 *v*/*v*/*v*), for DON: H_2_O/ACN/MeOH (94/3/3 *v*/*v*/*v*), for AFLB_1_, H_2_O/ACN/MeOH (60/20/30 *v*/*v*/*v*), for T-2 toxin: H_2_O/ACN (40/60 *v*/*v*); flow rate mL/min for all mycotoxins: 1 mL/min; injection volume for all mycotoxins: 100 μL; fluorescence detector, wavelength λ (nm) (excitation and emission) for AFLB_1_: 365 and 435; for T-2 toxin: 381 and 470; for ZEN: 274 and 418; UV detector λ (nm) for DON: 218. Limit of detection: AFLB_1_—0.2 μg/kg; ZEN—3 μg/kg; DON—20 μg/kg; T-2 toxin—1.4 μg/kg.

Chromatographic separation of mycotoxins was performed using a LiChrospher^®^ 100 RP-18 (Merck KGaA, Darmstadt, Germany), LiChroCART 250–4 column (250 mm × 4.0 mm, 5 μm; Supelco Park, Bellefonte, PA, USA). Mycotoxin concentrations were measured by comparing their retention times with the maximum retention times of standard solutions. Mycotoxin concentrations were determined by correlating the peak area of the samples with the standard curves obtained using HPLC analysis of standard solutions.

A 1290 Infinity ultra-high-performance liquid chromatography (UHPLC) system coupled with a 6460 triple quadrupole mass spectrometer (Agilent Technologies, Waldbronn, Germany) was utilized to quantify T-2 toxin in samples. The detection conditions were preoptimized for analysis. The separation was carried out using a Waters ACQUITY HSS T3 column (100 mm × 2.1 mm internal diameter, 1.8 μm particle size) from Waters (Milford, MA, USA) at a constant temperature of 40 °C. The mobile phase consisted of water with 0.1% formic acid, 5 mM ammonium formate (phase A), and methanol (phase B) according to González-Jartín et al. [24].

The elution gradient began with a 0.5-min hold at 0% B, followed by an increase to 14% B within 0.5 min, which was sustained for 1.5 min. The proportion of phase B then rose to 60% within 1 min and was held steady for 0.5 min. The gradient reached 100% B over 4.5 min and maintained this level for 2 min before returning to 0% B within 0.5 min, remaining at this level for 2.5 min. The flow rate was maintained at 0.3 mL/min, and a 5 μL sample injection volume was used.

The Agilent 6460 mass spectrometer was equipped with an electrospray ionization (ESI) source using Agilent Jet Stream Technology. The ion source parameters were as follows: capillary voltage at 4000 V, positive nozzle voltage at 1500 V, negative nozzle voltage at 0 V, nebulizer pressure at 45 psi, sheath gas flow at 12 L/min and 400 °C, and nebulizer gas flow at 8 L/min and 350 °C. For each mycotoxin, key parameters such as fragmentor voltage (FV), cell accelerator voltage (CAV), collision energy (CE), and mass transitions were optimized individually using MassHunter Optimizer software.

Performance characteristics for the T-2 toxin included: coefficient of variance (CV)—4.7%, matrix effect (SSE)—95.2%, apparent recovery (RA)—62.4%, precision (RSD)—5.6%, and accuracy (RE)—65.5%.

All mycotoxin determination methods are accredited in accordance with the LST EN ISO/IEC 17025 standard.

Limit of detection: AFLB_1_—0.2 μg/kg; ZEN—3 μg/kg; DON - 20 μg/kg; T-2 toxin—9.6 μg/kg.

Zearalenone metabolites in rumen samples from the static gastrointestinal model system were tested by gas chromatograph with a mass spectrometer and automatic sample entry system applying the electron mode method (GC-MS EI) (Agilent Technologies 6890N with automatic sample entry system 5975, Agilent, USA). The test was performed in a few steps: sample hydrolysis using Helix Pomatia with tert—butylmethylether, purification using columns Chromabond^®^ C_18_ (Macherey-Nagel GmbH & Co. KG, Düren, Germany) and Chromabond^®^ NH_2_ (Macherey-Nagel GmbH & Co. KG, Germany) for solid phase extraction. Metabolites were determined by adding 2 mL 2 mol/L acetate buffer, 25 μL Helix Pomatia, and 100 μL internal standard solution, mixing, and performing hydrolysis. After hydrolysis, 5 mL tert—butylmethylether was added, and after 5 min, the sample was for 10 min centrifuged at 3500 rpm (Centrifuge MPW—251, MPW, Warsaw, Poland). The extract was evaporated to a dry residue with a stream of nitrogen. The dry residue was dissolved in 3 mL MeOH and 1 mL 0.04 mol/L acetate buffer, then 2 mL n-hexane was added, and the mixture was centrifuged. After adding 5 mL dichloromethane, centrifuging was continued (Centrifuge MPW—251, MPW, Poland). After 20 min, the sample was evaporated using a stream of nitrogen. The obtained dry residue was dissolved in 5 mL of MeOH/H_2_O (40/60, *v*/*v*). The extract was passed through a 500 mg C_18_ column. The column was washed with MeOH/H_2_O (40/60, *v*/*v*) and acetone/water (20/80, *v*/*v*) and dried under weak vacuum conditions. The residue was dissolved in acetone/methanol (80/20, *v*/*v*), passed through a NH_2_ column, and evaporated to dry residue. GC-MS EI analysis parameters are presented in Table 4.

### 5.4. Statistical Analysis

The statistical analysis of data was performed using the IBM SPSS (version 25.0) statistical package (SPSS Inc., Chicago, IL, USA). To evaluate the effect of rumen pH environments (physiological (pH 6.5) and acidic (pH 5.5)) on AFLB_1_, ZEN, DON, and T-2 toxin mycotoxin concentrations, the data were analyzed using descriptive statistics and a one-way ANOVA. The differences in the test properties of the compared groups were expressed as mean values and the standard error of the mean (SEM), and differences between the rumen pH environments were assessed using Fisher’s LSD test (α = 5%). The obtained results were statistically significant when *p* < 0.05.

## Figures and Tables

**Table 1:** Percentage change of zearalenone concentration in studied mycotoxin combinations in physiological and acidic rumen conditions of dairy cows in vitro considering the duration of exposure (n = 25).

Zearalenone Combinations	Mycotoxins Concentrations in the Combinations, µg/L	Study Time, h	The Percentage Reduction of ZEN ± SEM	
			pH 6.5	pH 5.5
ZEN (n = 5)	1000	0 h	0 ^A^ ***	0 ^a^ ***
		3 h	36.0 ± 3.1 ^A^	3.4 ± 1.0 ^a,ABD^
		6 h	43.0 ± 2.0 ^A^ ***	3.8 ± 0.7 ^a,A^ ***
		9 h	44.0 ± 2.5 ^A^ ***	24.0 ± 1.0 ^b,A^ ***
ZEN + AFLB_1_ (n = 5)	1000 + 7	0 h	0 ^a,A^ ***	0 ^a^ ***
		3 h	48.0 ± 1.2 ^b^ ***	5.4 ± 1.3 ^b,B^ ***
		6 h	61.0 ± 1.0 ^c,B^ ***	10.0 ± 0.0 ^c,B^ ***
		9 h	77.0 ± 1.2 ^d,B^ ***	8.0 ± 1.2 ^bc,B^ ***
ZEN + DON (n = 5)	1000 + 600	0 h	0 ^a,B^	0 ^a^
		3 h	3.6 ± 0.7 ^b^ **	0.8 ± 0.5 ^b,C^ **
		6 h	48.6 ± 2.3 ^c,C^ **	40.0 ± 0.0 ^c,C^ **
		9 h	100 ± 0.0 ^d,C^ ***	38.4 ± 1.0 ^c,C^ ***
ZEN + T-2 (n = 5)	1000 + 500	0 h	0 ^a,B^	0 ^a^
		3 h	7.0 ± 5.8 ^a,B^	1.4 ± 1.0 ^a,DC^
		6 h	43.0 ± 2.0 ^b,A^ ***	22.6 ± 1.5 ^b,D^ ***
		9 h	50.5 ± 2.0 ^c,D^ ***	23.4 ± 1.4 ^b,A^ ***
ZEN + AFLB_1_ + DON + T-2 (n = 5)	1000 + 7 + 600 + 500	0 h	0 ^a,B^	0 ^a^
		3 h	24.6 ± 0.2 ^b^ ***	30.4 ± 0.4 ^a,DC^ ***
		6 h	25.4 ± 0.2 ^b,C^ ***	33.6 ± 0.7 ^a,A^ ***
		9 h	49.0 ± 1.0 ^c,C^ ***	46.6 ± 0.8 ^b,D^ ***

Note: ^a,b,c,d^—Means within a column (for each mycotoxin combination) marked with different letters differ significantly (*p* < 0.05, Fisher’s LSD test); ^A,B,C,D^—Means within a column (different time points: 0, 3, 6, and 9 h) marked with different letters differ significantly (*p* < 0.05); **—*p* < 0.01; ***—*p* < 0.001—indicate the statistical significance of the difference between pH 6.5 and pH 5.5 groups, Student’s *t*-test for independent samples.

**Table 2:** Percentage change of T-2, AFLB_1_, and DON concentrations in combination with ZEN in physiological and acidic rumen conditions of cattle in vitro considering the duration of exposure (n = 25).

Mycotoxins Combinations	Mycotoxins Concentrations in the Combinations, µg/L	Study Time, h	The Percentage Reduction of AFLB_1_ ± SEM		The Percentage Reduction of DON ± SEM		The Percentage Reduction of T-2 ± SEM	
			pH 6.5	pH 5.5	pH 6.5	pH 5.5	pH 6.5	pH 5.5
ZEN + AFLB_1_ (n = 5)	1000 + 7	0 h	0 ^a^	0 ^a^				
		3 h	31.4 ± 7.0 ^b,B^	15.7 ± 5.2 ^b,B^				
		6 h	52.9 ± 2.9 ^c,B^ ***	14.3 ± 0.0 ^b,B^ ***				
		9 h	85.7 ± 9.0 ^d,B^ **	25.7 ± 2.9 ^c,B^ **				
ZEN + DON (n = 5)	1000 + 600	0 h			0	0 ^a^		
		3 h			18.7 ± 1.3 ^b,B^ ***	18.3 ± 4.1 ^a,A^ ***		
		6 h			60.7 ± 1.6 ^c,B^	30.0 ± 2.1 ^b,B^		
		9 h			98.3 ± 1.7 ^bc,B^	63.3 ± 2.0 ^c,A^		
ZEN + T-2 (n = 5)	1000 + 500	0 h					0 ^B^	0 ^a,A^
		3 h					55.0 ± 1.6 ^B^ *	50.0 ± 0.0 ^a,A^ *
		6 h					56.0 ± 1.0 ^B^ *	46.0 ± 2.4 ^a,B^ *
		9 h					58.0 ± 2.0 ^B^	56.0 ± 2.4 ^b,B^
ZEN + AFLB_1_ + DON + T-2 (n = 5)	1000 + 7 + 600 + 500	0 h	0 ^a^	0 ^a^	0 ^a^	0 ^a^	0 ^a,C^ ***	0 ^a,B^ ***
		3 h	81.4 ± 1.7 ^b,C^ ***	25.7 ± 2.9 ^b,C^ ***	60.0 ± 1.7 ^C^	1.3 ± 0.8 ^b,B^	34.4 ± 3.9 ^a,C^ ***	2.0 ± 2.0 ^a,B^ ***
		6 h	89.1 ± 0.9 ^b,C^ ***	25.7 ± 2.9 ^b,C^ ***	75.0 ± 5.3 ^C^ ***	60.0 ± 1.7 ^c,C^ ***	56.0 ± 2.4 ^b,C^ ***	38.4 ± 1.6 ^b,C^ ***
		9 h	91.1 ± 0.7 ^c,C^	90.6 ± 0.6 ^c,C^	70.0 ± 2.0 ^C^ ***	68.3 ± 1.7 ^d,A^ ***	50.0 ± 5.0 ^b,C^	31.2 ± 6.6 ^b,C^

Note: ^a,b,c,d^—Means within a column (for each mycotoxin combination) marked with different letters differ significantly (*p* < 0.05, Fisher’s LSD test); ^A,B,C^—Means within a column (different time points: 0, 3, 6, and 9 h) marked with different letters differ significantly (*p* < 0.05); *—*p* < 0.05; **—*p* < 0.01; ***—*p* < 0.001—indicate the statistical significance of the difference between pH 6.5 and pH 5.5 groups, Student’s *t*-test for independent samples.

**Table 3:** Ingredients (percentage) of the total mixed ration (TMR) ration given to rumen fluid donor dairy cows used to test in an in vitro rumen model.

TMR Components	Percentage of Ration
Grass silage (haylage)	40
Maize silage	18
Barley and wheat flour	12
Straw	2.5
Compound feed ^1^	27.5
**Chemical composition of feeding rations, % DM**	
DM	49.2
CP	16.2
NDF	36.2
ADF	19.6

Note: DM—dry matter; CP—crude protein; NDF—neutral detergent fiber; ADF—acid detergent fiber, TMR—total mixed ration, ^1^ Ingredients of compound feed: cereal grains (wheat, triticale, oats, and barley), rape seed expeller, soya meal, soda, feed lime, salt, molasses, vitamins, and mineral feed supplements.

**Table 4:** Gas chromatograph with mass spectrometer and automatic sample entry system analysis parameters.

Parameters	Zearalenone Metabolites	
	Alpha-Zearalenol (α-ZEL)	Beta-Zearalenol (β-ZEL)
Stationary phase layer (μm)	0.25	0.25
Injection, in splitless regime (μL)	1	1
Temperature of automatic sample entry system (°C)	260	260
Temperature of MS ion source/temperature of MS quadrupole (°C)	230/150	230/150
Line velocity of stream speed (cm/s)	46	46
Limit of detection (LOD), (μg/L)	0.04	0.05

## Data Availability

The original contributions presented in this study are included in the article. Further inquiries can be directed to the corresponding author(s).
